# Supplementary material for: Establishing the Link between X-Chromosome Aberrations and TP53 Status, with Breast Cancer Patient Outcomes
Source: Cells. 2023 Sep 11;12(18):2245. doi: 10.3390/cells12182245 (PMC10526523; doi:10.3390/cells12182245)
Supplement: Supplementary file 1 [file cells-12-02245-s001.zip › Figure S1.pdf]

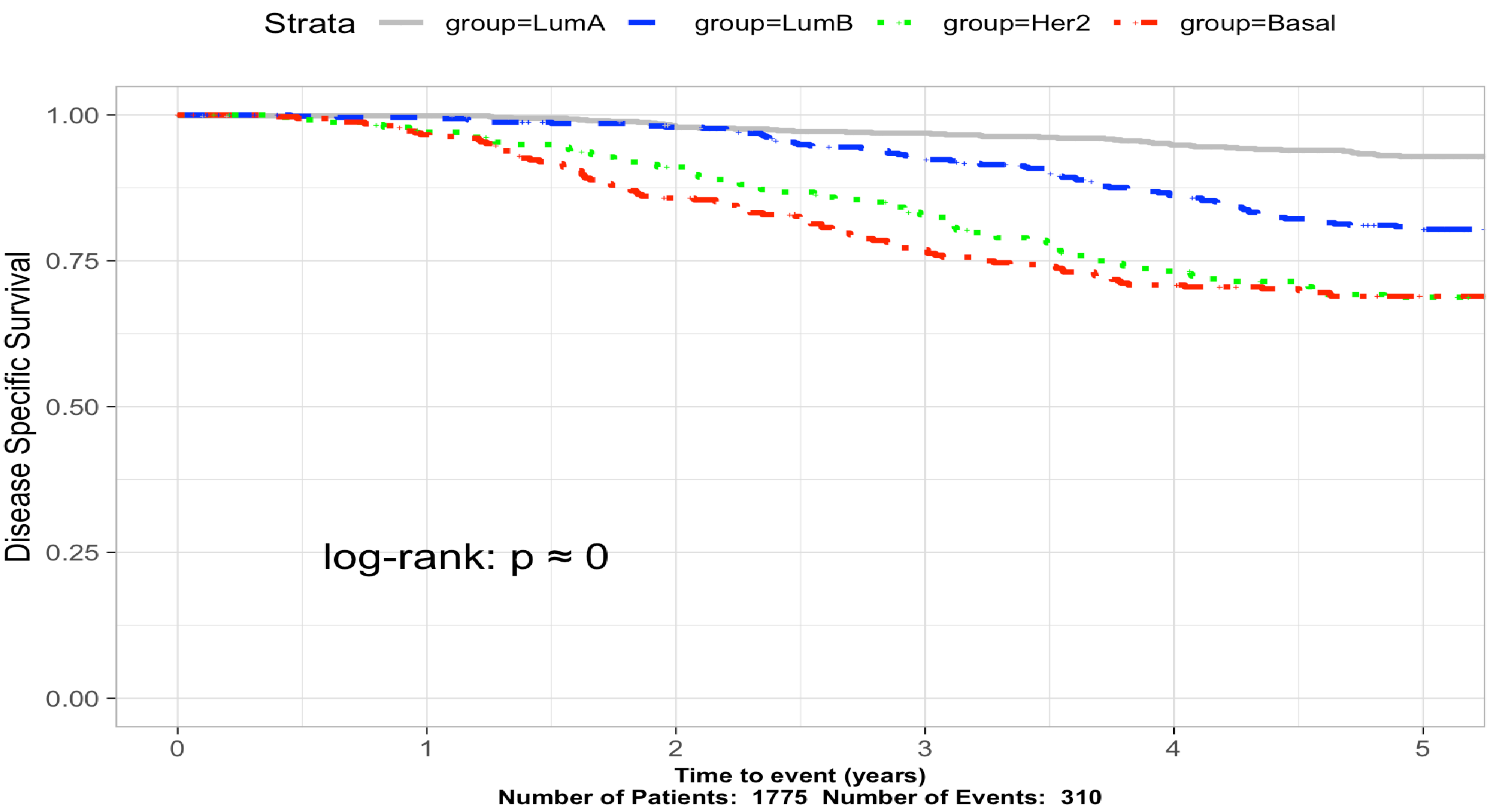

Supplementary Figure S1: Survival analysis of the four most frequent PAM50 molecular subtypes in the METABRIC cohort.
