# Supplementary figures and images for: Establishing the Link between X-Chromosome Aberrations and TP53 Status, with Breast Cancer Patient Outcomes
Source: Cells. 2023 Sep 11;12(18):2245. doi: 10.3390/cells12182245 (PMC10526523; doi:10.3390/cells12182245)

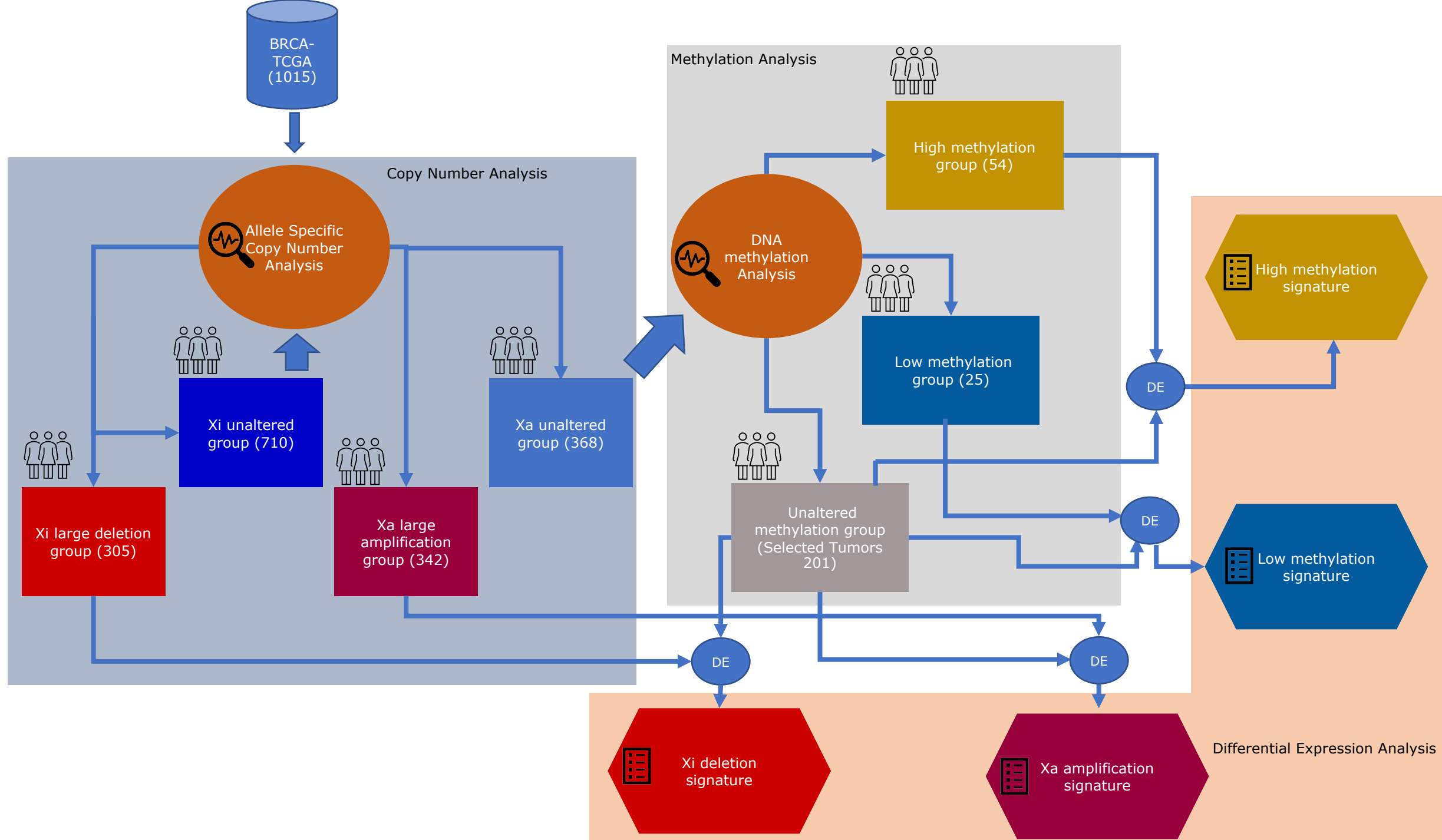

Supplementary Diagram S1: Analysis pipeline of BRCA-TCGA samples.

Supplement: Supplementary file 1 [file cells-12-02245-s001.zip › Diagram S1.pdf]

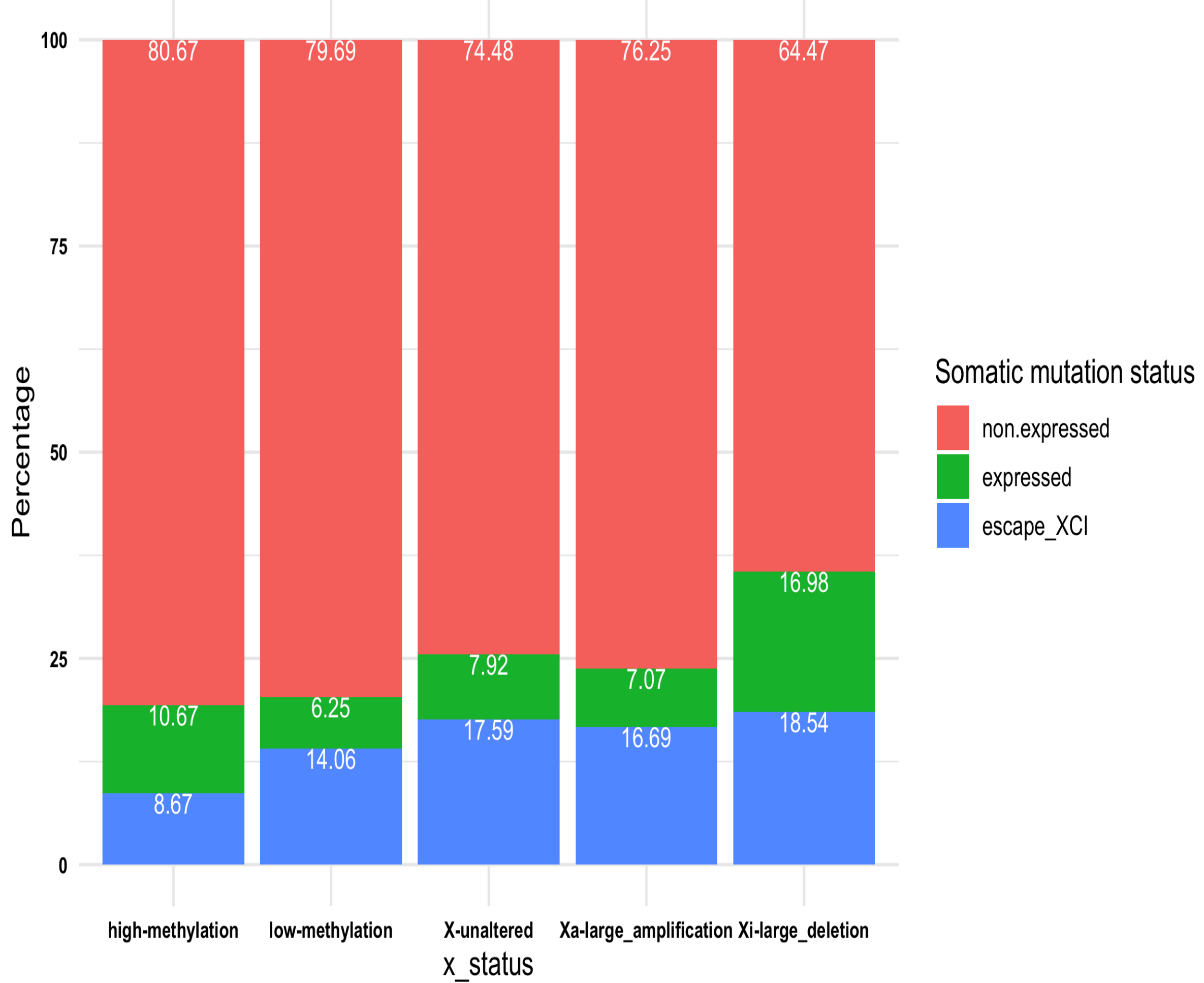

**Supplementary Figure S3**

Supplement: Supplementary file 1 [file cells-12-02245-s001.zip › Figure S3.pdf]

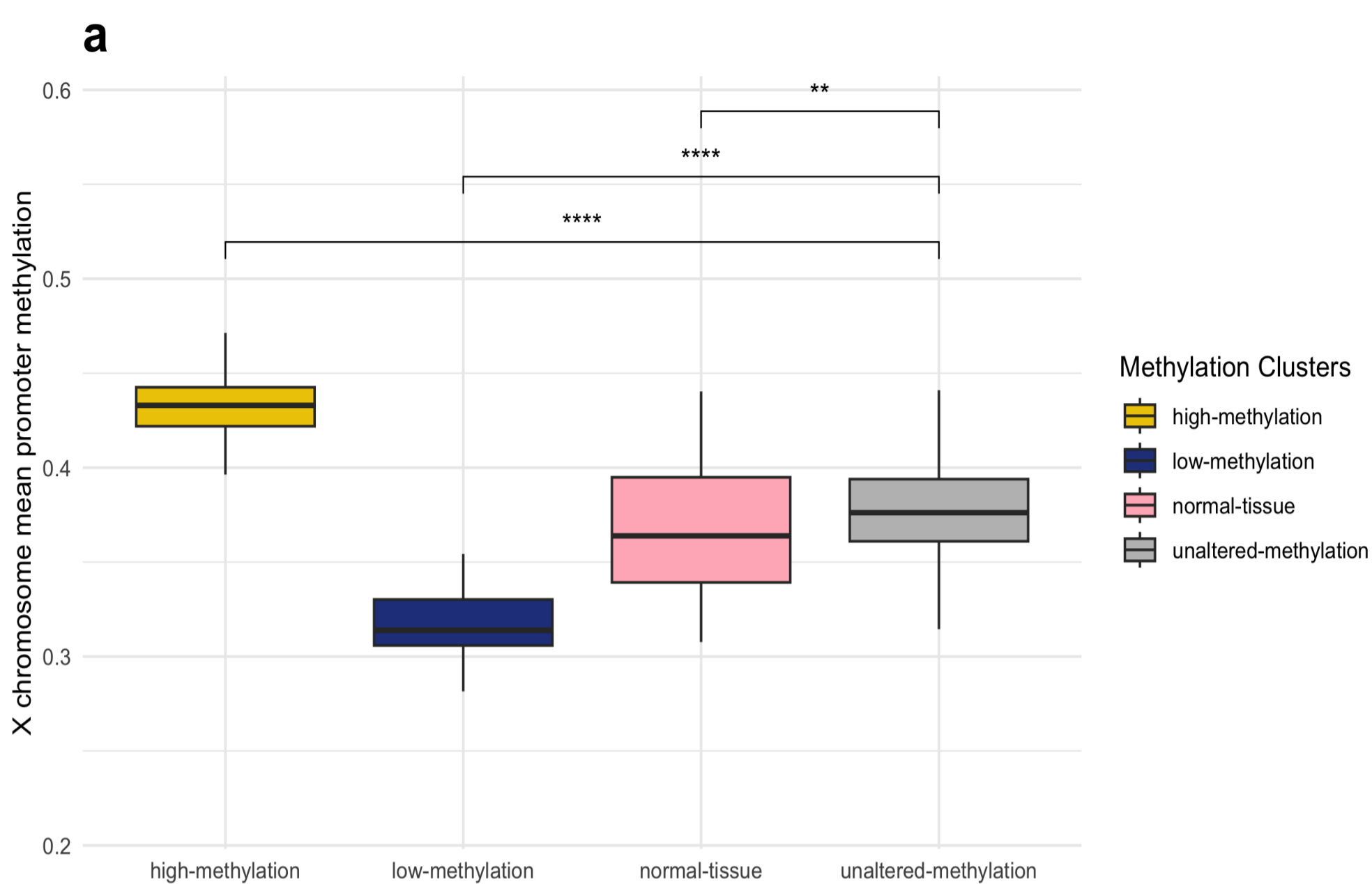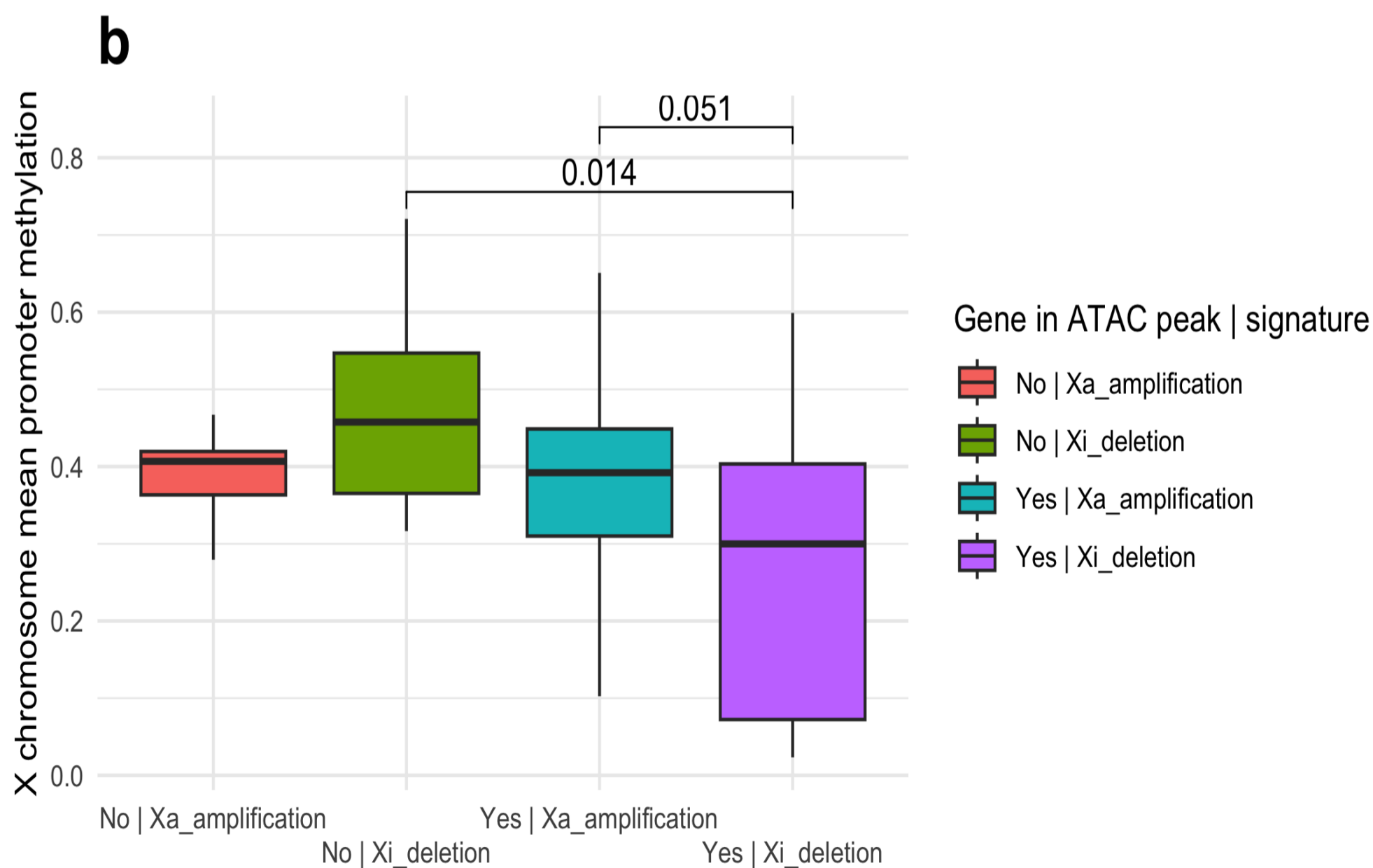

**Supplementary Figure S4**

Supplement: Supplementary file 1 [file cells-12-02245-s001.zip › Figure S4.pdf]
